# Quantitative evaluation of macromolecular crowding environment based on translational and rotational diffusion using polarization dependent fluorescence correlation spectroscopy

**DOI:** 10.1038/s41598-021-89987-7

**Published:** 2021-05-19

**Authors:** Johtaro Yamamoto, Akito Matsui, Fusako Gan, Makoto Oura, Riku Ando, Takahiro Matsuda, Jian Ping Gong, Masataka Kinjo

**Affiliations:** 1 Bioimaging Research Group, Health and Medical Research Institute, National Institute of Advanced Industrial Science and Technology (AIST), Tsukuba, 305-8566 Japan; 2 Laboratory of Molecular Cell Dynamics, Faculty of Advanced Life Science, Hokkaido University, Sapporo, 001-0021 Japan; 3 Graduate School of Life Science, Hokkaido University, Sapporo, 001-0021 Japan; 4 Laboratory of Soft & Wet Matter, Faculty of Advanced Life Science, Hokkaido University, Sapporo, 001-0021 Japan; 5 Institute for Chemical Reaction Design and Discovery (WPI-ICReDD), Hokkaido University, Sapporo, 001-0021 Japan; 6 Global Institution for Collaborative Research and Education (GI-CoRE), Hokkaido University, Sapporo, 001-0021 Japan

**Keywords:** Applied optics, Biophysics, Nanoscale biophysics, Polymer characterization

## Abstract

Macromolecular crowding (MMC) in cells is a hot topic in biology; therefore, well-characterized measurement standards for the evaluation of the nano-environment in MMC solutions are necessary. We propose to use polarization-dependent fluorescence correlation spectroscopy (Pol-FCS) for evaluation of macromolecular crowding in solutions. Pol-FCS can simultaneously measure the relaxation times of rotational and translational diffusion of fluorescent molecules at the same position, even in living cells with low damage. In this report, the differences in the nano-environment among solutions of small molecules, gels, and MMC solutions were evaluated by comparing their rotational and translational diffusion using Pol-FCS. Moreover, this method could distinguish the phase shift in the polyethylene glycol solution. Finally, we separately evaluated the nano-environment in the cytosol and nucleus of living cells in different cell lines and cell cycles. We expect this evaluation method to be useful in characterizing the nano-environment in MMC studies. In addition, the proposed method may be useful for other nano-environments such as liquid–liquid phase separation.

## Introduction

Recently, the effect of macromolecular crowding (MMC) on intermolecular interaction^1–3^, molecular structure, thermal stability^4^, and diffusion^5–8^ has become an attractive research target in biology and biophysics^9,10^. A large number of proteins, nuclear acids, and large structures such as organelles occupy about 20–30% of the cell volume^9^; thus, the intermolecular interaction and molecular conformation in such an environment is different from that in a lysate or solution. These differences between in vivo and in vitro measurements are because of the effect of MMC in such cells.

Most studies on MMC use certain chemical substances called crowders or crowding agents, such as polyethylene glycol (PEG), ficoll, and bovine serum albumin (BSA), to mimic MMC conditions in cells. Some measurements are compared with crowder-abundant media and solutions without crowders. The activity coefficients of molecules in crowding conditions are generally increased by the size exclusion effect of crowders, and the diffusion coefficients of molecules are decreased through collision with the surrounding crowders. MMC, therefore, has a complicated relationship with the reaction rates of molecules^9^.

As mentioned, MMC can be useful in understanding biochemical reactions in cells; however, there are no standard methods to evaluate the degree of crowding. In most studies, MMC is defined only by the concentration of crowders; however, this concentration does not reflect the network structure, such as the size and shape of the intermolecular gaps in the crowders.

One popular method in this context is sedimentation equilibrium centrifugation^11^. This method measures thermodynamic activity rather than the MMC environment. It indirectly provides MMC properties by measuring the activity of molecules labeled with fluorescent or radioactive tags in an MMC solution.

A method to characterize the MMC environment is to analyze the anomalous diffusion property of probe molecules in MMC solutions. It was reported that the diffusion of proteins was subdiffusive in solutions crowded by dextran using fluorescence correlation spectroscopy (FCS) with an anomalous diffusion model^12^. Masuda et al. developed a sampling-volume-controlled FCS to analyze anomalous diffusion or anomalous transport. They successfully measured the anomalous diffusion property of organic fluorescent dye in the hyaluronan aqueous solutions with various sufficient confocal volumes to obtain diffusion properties at various scales^13^. Wawrezinieck et al. introduced the concept of FCS diffusion law, which is the translational diffusion time dependency on the spatial scale defined by beam waist of focal volume^14^. The beam waist was controlled by changing diameter of excitation laser incident to the back aperture of the objective lens. They found that the FCS diffusion laws are strongly related to property of confined diffusion from simulations and experiments on a raft marker and transmembrane protein on cell membrane. Similar analysis was carried out on a membrane marker in supported phospholipid bilayers using Z-scan FCS in which the measurement area was controlled by shifting the sample plane from focal plane in z-axis^15^.

A potential way to characterize the MMC environment is to compare the translational diffusion and rotational diffusion of probe molecules. It was reported that the translational and rotational diffusion coefficients of molecules in an MMC solution deviate from the values predicted using Stokes–Einstein law and Stokes–Einstein–Debye law. Li et al*.* compared the rotational diffusion and translational diffusion of chymotrypsin inhibitor 2 (CI2) in two types of bulk solutions, glycerol and polyvinylpyrrolidone (PVP), using nuclear magnetic resonance spectroscopy (NMR)^7^. They found that the translational diffusion coefficient showed a negative deviation from the predicted value, and this negative deviation was greater for rotational diffusion in the PVP solution. Lee et al*.* also investigated the translational and rotational diffusion of fluorescent molecules in MMC solution using FCS and time-resolved fluorescence anisotropy (TRFA)^6^. They revealed the deviation in the translational and rotational coefficients from the predicted value in the ficoll solution and its dependency on the size of the probe molecule. Swaminathan et al*.* compared the translational diffusion measured by fluorescence recovery after photobleaching (FRAP) and the rotational diffusion measured by time-resolved fluorescence in living cells^16^. They found that the rotation of GFP in the cytosol was 1.5-fold slower than that in nonviscous saline solution, and the translation of GFP was ~ 3.2-fold slower than that in water. Polarized FCS based on a pulsed excitation laser and a time-correlated single-photon counting (TCSPC) instrument was developed to measure rotational diffusion and translational diffusion simultaneously by Loman et al.^17^. Using the polarized FCS, Roos et al. analyzed the diffusion property of the crowder itself^18^.

Another approach to FCS using mean squared displacement was also proposed^19^. This report analyzed the cell-size confinement effect in addition to the MMC effect.

Thus, comparing the translational diffusion and rotational diffusion of probe molecules is expected to characterize the MMC environment. However, the translational diffusion and rotational diffusion are measured by different methods, except for polarized FCS. This means translational diffusion and rotational diffusion are measured at different positions and times. Such measurements are not suitable for highly heterogeneous samples, including cells. Thus, methods that enable simultaneous measurement of translational diffusion and rotational diffusion at the same position are required.

We recently constructed a polarization-dependent fluorescence correlation spectroscopy (Pol-FCS) based on a continuous excitation and a hardware correlator^20,21^. Pol-FCS was proposed by Ehrenberg and Rigler in 1974^22–25^. However, Pol-FCS has not been used for live cell measurement because of the insufficiency of the performance of instruments (sensitivity of detector and time resolution of the hardware correlator). In 2016, we succeeded in the simultaneous measurement of the translational and rotational diffusion of green fluorescent protein (GFP) in living cells^21^. The results showed that the translational diffusions in the cytosol or nucleus were three times slower than those in phosphate-buffered saline (PBS), while the rotational diffusions were only two times slower, which was consistent with the results of FRAP and time-resolved fluorescence^16^.

In this report, we demonstrated the applicability of Pol-FCS to the existing evaluation method of the MMC environment based on the comparison between the translational diffusion and rotational diffusion of probe molecules. Pol-FCS measurements were performed on GFP in various solutions with and without crowders and macromolecular gels to demonstrate the possibility that Pol-FCS can characterize MMC solutions based on the translational and rotational diffusion of GFP molecules. Finally, we characterized the MMC environment in cells using Pol-FCS and observed their differences in some cell lines and some cell cycles.

## Results

In this study, we used GFP as a probe for the MMC environment. GFP is the most studied fluorescent protein, and its structure has also been solved. The shape of GFP is a quasi-cylindrical with a Stokes radius of 2.82 nm^26^, which is approximately 100 times smaller than the lateral radius of the measurement volume of our Pol-FCS system (237.9 nm). It has already been confirmed that the chromophore unit of GFP is rigidly linked to the barrel structure by time-resolved anisotropy decay experiments; therefore, the wobbling effect of chromophores is negligible^27^. Moreover, we can expect that the dynamics of GFP in mammalian cells would not be affected by its specific associations with surrounding proteins.

**Figure 1:**
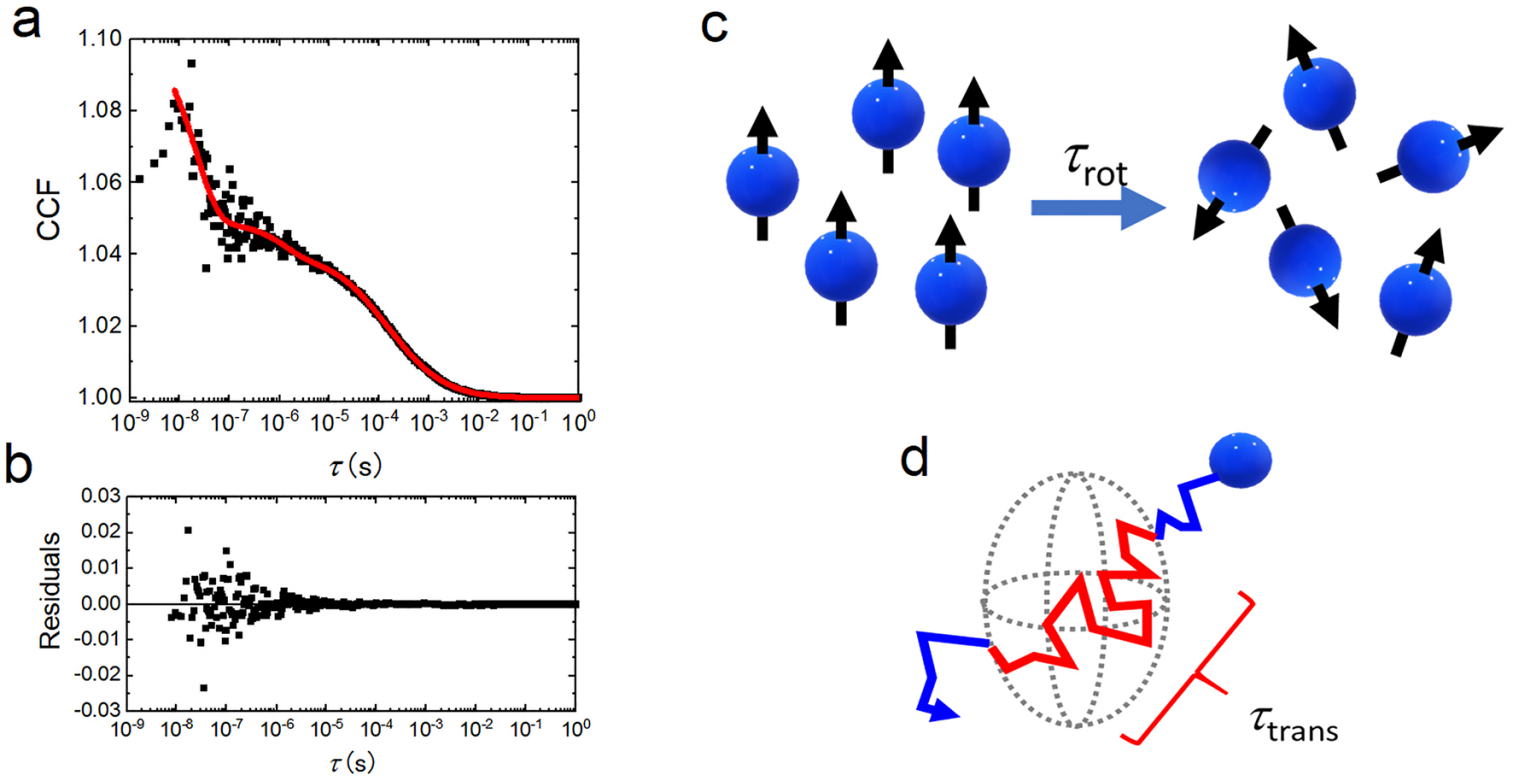
Typical cross-correlation function (CCF) of Pol-FCS measurement. (**a**) Typical CCF of 100 nM GFP in PBS. (**b**) Residuals of fitting analysis. (**c**) Schematic diagram of rotational diffusion time. (**d**) Schematic diagram of translational diffusion time.

Figure 1a shows a typical cross-correlation function of 100 nM GFP in PBS obtained by a Pol-FCS measurement. This was just a control experiment to confirm the Pol-FCS system. A similar experiment was already reported in 1999, and the results are in good agreement with the results of Fig. 1^27^.

The rapidly decaying component around 10^–8^–10^–7^ s is a rotational diffusion component, and its characteristic decay time (rotational diffusion time) is *τ*_rot_ = 22.5 ± 1.4 ns (average ± standard error, *n* = 6). The decay component around 10^–5^–10^–3^ s is the translational diffusion component, and its characteristic decay time (translational diffusion time) is *τ*_trans_ = 180.1 ± 12.4 μs (*n* = 6). Rotational diffusion time is the time required by target molecules to change from an initial orientation to a randomized orientation through rotational diffusion (Fig. 1c), and translational diffusion time corresponds to the average dwell time of the target molecule in the measurement volume (Fig. 1d). Longer translational/rotational diffusion times therefore indicate slower translational/rotational diffusions.

Cells, such as a cytoskeleton, have an immobile structure. First, we adopt chemically crosslinked hydrogels as a model system to study the effect of immobile environment on the diffusion of GFP. We adopted polyzwitterionic hydrogels consist of poly-*N*-(carboxymethyl)-*N*,*N*-dimethyl-2-(methacryloyloxy) ethanaminium inner salt (pCDME) in this work since polyzwitterions are less adhesive to proteins^28^. The mesh size ξ of the gels is tuned by varying the crosslinker density during gel preparation.

**Figure 2:**
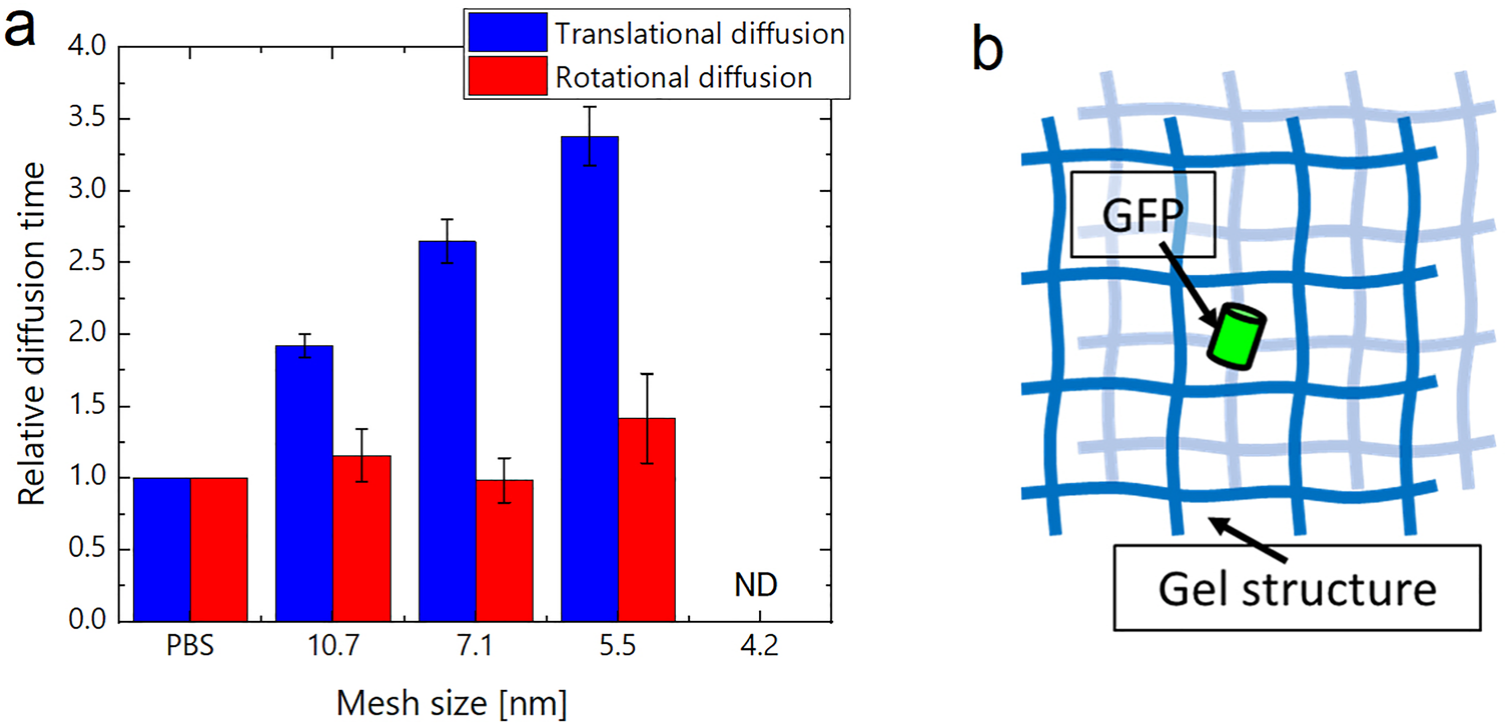
Pol-FCS measurement of GFP in gels. (**a**) Relative rotational/translational diffusion times. The sample with the mesh size of 4.2 nm could not be measured because GFP could not enter the gel. (**b**) GFP in gel structure.

Figure 2a shows a comparison of the translational/rotational diffusion times of GFP between PBS and gels soaked in PBS. Here, we have used the relative translational/rotational diffusion times *τ*_tans_′ and *τ*_rot_′, which are the ratios of those values in samples to those in PBS as reference samples, and are defined as

1 $${\tau {^{\prime}}}_{\text{rot}}=\frac{{\tau }_{\text{rot}}}{{\tau }_{\mathrm{rot},0}}$$

2 $${\tau {^{\prime}}}_{\mathrm{trans}}=\frac{{\tau }_{\mathrm{trans}}}{{\tau }_{\mathrm{trans},0}}$$

where *τ*_rot,0_ and *τ*_trans,0_ are the rotational and translational diffusion times of the reference sample, respectively.

The translational diffusion time increases with the decrease in the mesh size above a threshold value while the rotational diffusion is hardly influenced by the mesh size ξ. The threshold mesh size around ~ 4 nm is consistent with the GFP size, indicating that only when the mesh size of the gels is larger than the probe size, it can diffuse inside the gels, as shown in Fig. 2b.

**Figure 3:**
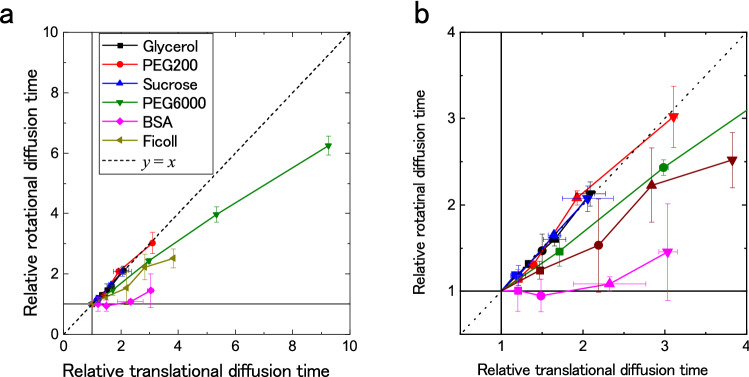
Comparison between the translational and rotational diffusion times normalized by these values of GFP solution in PBS. (**a**) Whole plot. (**b**) Enlarged plot. The error bars show standard deviations (*n* = 3). Pol-FCS measurement for 30 s was repeated 10 times. The excitation laser power was 3.2 µW (1.6 kW/cm^2^) at the samples. The concentrations of each sample at each data point are 0, 50, 100, 150, and 200 mg/mL in order from point (1, 1) for each crowder.

Next, we evaluated the MMC environment based on Pol-FCS. Figure 3 shows the relationship between relative translational diffusion time and relative rotational diffusion time. Measurements were performed on GFP in uniform viscous solutions and MMC solutions of glycerol (Wako, Japan), sucrose (Nacalai Tesque, Japan), ficoll (Nacalai Tesque, Japan), polyethylene glycol 6,000 (Wako, Japan) (PEG6,000), polyethylene glycol #200 (PEG200) (Nacalai Tesque, Japan), and BSA (Sigma-Aldrich, USA).

The translational diffusion time and rotational diffusion time for spherical particles can be expressed as

3 $${\tau }_{\mathrm{trans}}=\frac{{w}_{0}^{2}}{4{D}_{\mathrm{trans}}}$$

4 $${\tau }_{\mathrm{rot}}=\frac{1}{6{D}_{\mathrm{rot}}}$$

where *w*_0_ is the lateral radius of the measurement volume. *D*_trans_ and *D*_rot_ are the translational diffusion coefficient and the rotational diffusion coefficient, respectively^21,23,29,30^. According to the Stokes–Einstein equation and Einstein–Debye equation, both *D*_trans_ and *D*_rot_ are inversely proportional to the viscosity of the medium as follows:

5 $${D}_{\mathrm{trans}}=\frac{{k}_{B}T}{6\pi \eta r}$$

6 $${D}_{\mathrm{rot}}=\frac{{k}_{B}T}{8\pi \eta {r}^{3}}$$

where *k*_*B*_, *T*, *η*, and *r* are the Boltzmann constant, absolute temperature, viscosity, and hydrodynamic radius of the particle, respectively. Thus, the relative rotational and translational diffusion times are

7 $${\tau {^{\prime}}}_{\mathrm{rot}}={\tau {^{\prime}}}_{\mathrm{trans}}=\frac{\eta }{{\eta }_{0}}$$

where *η*_0_ is the viscosity of the reference sample. The relative rotational diffusion time is proportional to the relative translational diffusion time in a uniformly viscous media. In that case, the data plots should be on the line *y* = *x*, and the results of glycerol, sucrose, and PEG200 would be consistent. However, the results from the other MMC solutions were not on this line.

**Figure 4:**
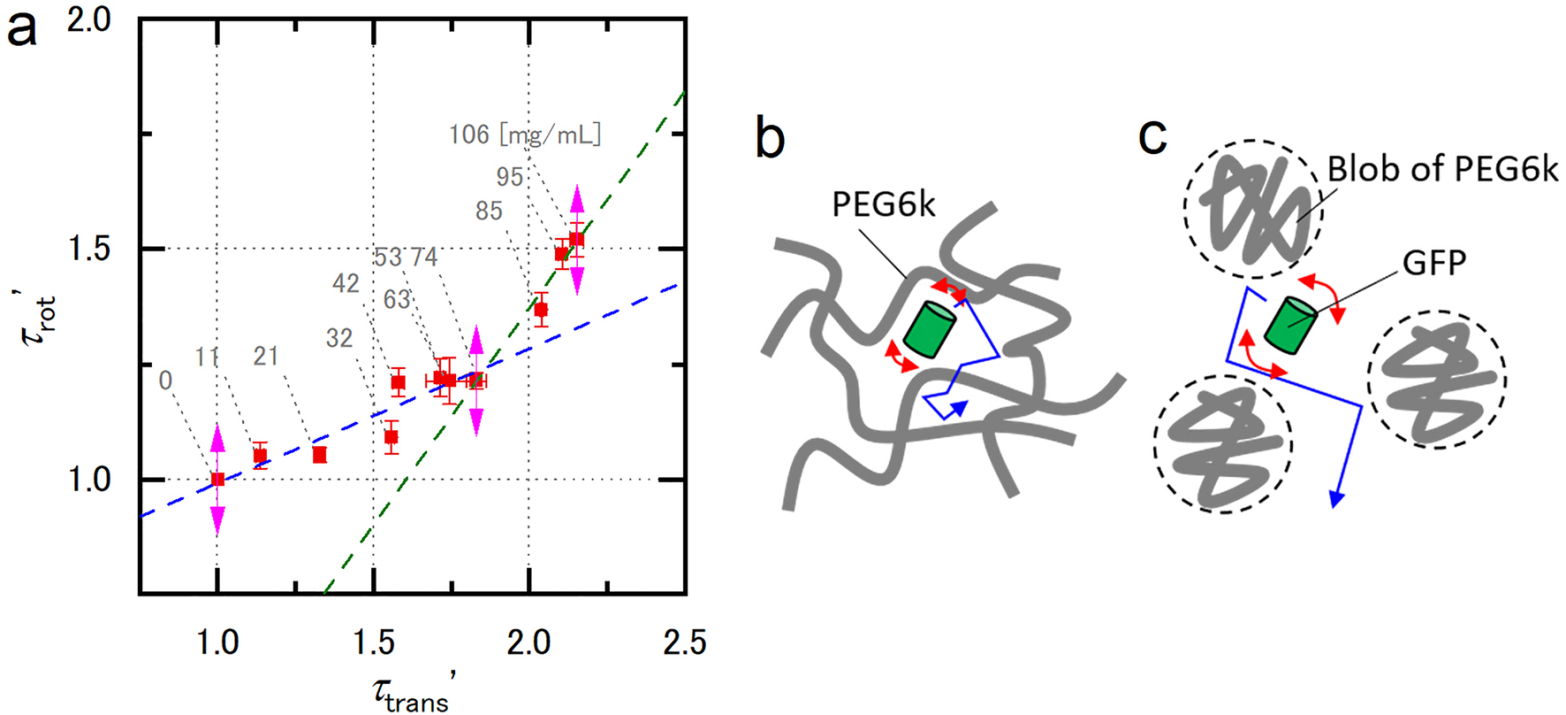
Results of PEG6,000 around its overlap concentration (Estimated c* was 79 mg/mL). (**a**) Comparison between the relative translational and rotational diffusion times normalized by these values of GFP solution in PBS. The error bars show standard deviations (*n* = 3). Pol-FCS measurement for 30 s was repeated 10 times. The excitation laser power was 6.5 µW (3.3 kW/cm^2^) at the samples. The concentrations of each sample at each data point were 0, 11, 21, 32, 42, 53, 63, 74, 85, 95, and 106 mg/mL in order from point (1, 1) for each crowder. The blue broken line and green broken line show the linear regression lines in the range of $1.00\le {\tau }_{\text{trans}}^{\prime}\le 1.83$ and $1.83\le {\tau }_{\text{trans}}^{\prime}\le 2.15$, respectively. The blue broken line is ${\tau }_{\text{rot}}^{\prime}=0.29{\tau }_{\text{trans}}^{\prime}+0.70$. The green broken line is ${\tau }_{\text{rot}}^{\prime}=0.94{\tau }_{\text{trans}}^{\prime}-0.51$. (**b**) Schematic diagram of the estimated nano-environment in the sample with concentration lower than c*. (**c**) Schematic diagram of the estimated nano-environment in the sample with concentration higher than c*. Red and blue arrows show the rotation and translation of GFP.

A more detailed analysis was performed on the PEG6,000 solution. The results are shown in Fig. 4a. The slope of the plots clearly changed at a concentration of 74 mg/mL. This change is the result of a phase change at the overlap concentration of PEG6,000 from a blob (Fig. 4b) to the entangled structure (Fig. 4c). The smaller standard deviation compared to that in Fig. 3 is likely due to the improvement of the signal-to-noise ratio by increasing the excitation laser power.

**Figure 5:**
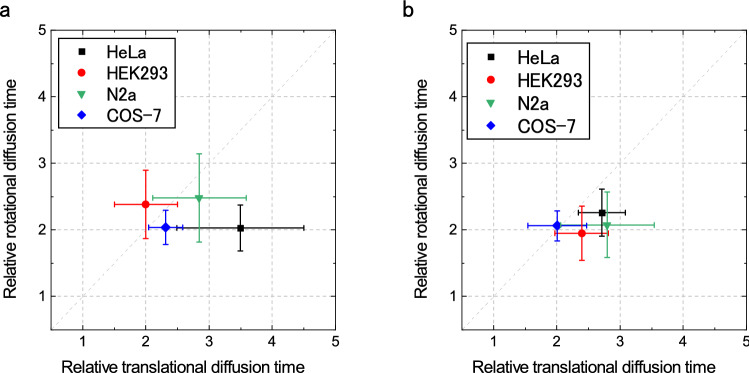
Pol-FCS result of GFP in different cell lines. The translational/rotational diffusion times were normalized by these values of GFP solution in PBS. (**a**) Results without nocodazole treatment. (**b**) Results with nocodazole treatment. The excitation laser power was 11 µW (5.6 kW/cm^2^). The measurement duration was 100 s (= 5 s × 20 loop). Error bars show standard deviation (*n* = 14–20).

In order to demonstrate the applicability of Pol-FCS to live cell measurements, Pol-FCS measurements of GFP were performed in the cytosol of different cell lines, HeLa, HEK293, Neuro 2a (N2a), and COS-7 with/without nocodazole treatment. It is known that nocodazole inhibits tubulin polymerization and quickly depolymerizes microtubules in cultured cells^31,32^. The results are shown in Fig. 5. Figure 5a shows the results without nocodazole treatment. Relatively large standard deviations, likely due to the highly heterogeneous environment inside cells and individuality of the cells, were observed. All average value and standard deviation are shown in Supplementary Tables S1 and S2, and *p*-values obtained by Welch’s *t*-test are shown in Supplementary Tables S3–S7. There were statistical significant differences among the cell lines without nocodazole treatment, especially in translational diffusion (See Supplementary Table S3). The difference of rotational diffusion was smaller than the translational diffusion (See Supplementary Table S4). The most of significant differences of the translational diffusion and the rotational diffusion disappeared by nocodazole treatment (see Supplementary Tables S5 and S6). Supplementary Table S7 show the effect of nocodazole treatment on the translational diffusion and the rotational diffusion in cytosol of each cell line. The effect was likely different for the different cell lines.

**Figure 6:**
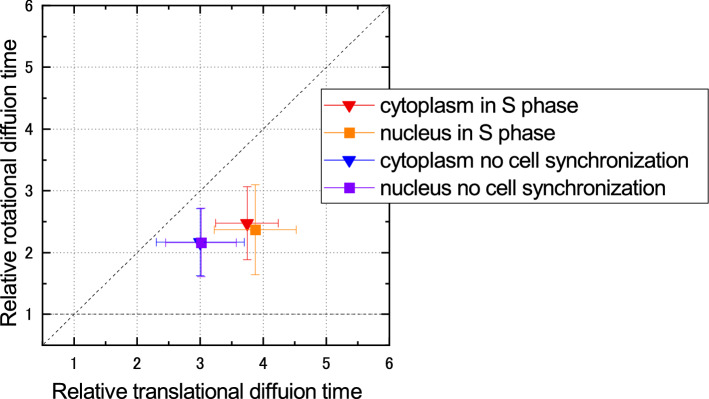
Pol-FCS result of GFP in living cells. The translational/rotational diffusion times were normalized by these values of GFP solution in PBS. The samples were HeLa cells expressing GFP with/without cell cycle synchronization at S phase. Error bars show standard deviation (*n* = 20–23).

Finally, the MMC condition in living cells was analyzed to demonstrate the in vivo measurement of Pol-FCS. The sample was HeLa cells expressing GFP. Measurements were performed in the cytosol and nucleus with/without cell cycle synchronization at the S phase using the double thymidine block method. Figure 6 shows the relative diffusion time plot. The relative translational diffusion time ranged from 3 to 4. The relative rotational diffusion time ranged from 2 to 2.5. This result agrees well with earlier reports^16,21^. All the data for Fig. 6 and the results of Welch’s *t*-test are shown in Supplementary Tables S8 and S9. It was shown that there were no difference of the rotational/translational diffusion between cytosol and nucleus in HeLa cells with/without cell cycle synchronization at S phase. The statistical significant differences of translational diffusion in both the cytosol and nucleus were found between with/without cell cycle synchronization.

## Discussions

In this study, we demonstrated the applicability of Pol-FCS to the existing evaluation method of the MMC environment based on the comparison between the translational diffusion and rotational diffusion of probe molecules. Pol-FCS can measure rotational and translational diffusion at the same spatiotemporal position (confocal volume). This is the most important advantage of Pol-FCS because the nano-environment is not uniform in MMC samples and its distribution is important for understanding MMC especially in cells. If the rotational and translational diffusion measurements are performed at different positions, the nano-environment would not be understood correctly.

First, it was comfirmed that the rotational diffusion time of GFP can be successfully measured by our Pol-FCS system (Fig. 1). The rotational diffusion time was 22.5 ns, which agrees well with the reported values^6,27,33^.

The results in Fig. 2 show that the smaller mesh size of gels made the translational diffusion time longer than that in a solution, indicating that the mesh structure of gels disturbed the translational diffusion of GFP. In contrast, the rotational diffusion time did not change. This result suggests that the rotational diffusion was not disturbed by the mesh-like structure of the gels because the GFP is difficult to move translationally in several tens of nanoseconds of rotational diffusion time. If the rotational radius is sufficiently shorter than the mesh size of the gels, the rotational diffusion time would not change. This result also indicates the possibility of mesh size estimation based on the ratio between the relative translational and rotational diffusion times. Similarly, the estimation of gap size among crowders in an MMC solution would be possible.

When measured by Ostwald viscometers, the viscosity of the gels would be infinite. However, the GFP in gels could be diffused, and its translational and rotational diffusion was differently affected by the mesh structure of the gels. This means that the viscosity is not the same in different spatial/temporal scales. Determining the viscosity of an environment around a target molecule is difficult. Therefore, the viscosity parameter is not suitable for analyzing this study. On the other hand, comparisons between viscosity and translational/rotational diffusion are usually performed in MMC studies.

The rotational and translational diffusion in the MMC solutions are compared in Fig. 3. The plots of the solution of small molecules (glycerol, sucrose, and PEG200 solutions) are on the line *y* = *x*, indicating that the rotational and translational diffusion in such solutions obey the Stokes–Einstein equation and Einstein–Debey equation. In contrast, a bulky crowder like BSA can disturb the translational diffusion but cannot disturb the rotation of GFP because the plots were around *y* = 1. PEG6,000 and ficoll disturbed both translational diffusion and rotational diffusion. In this case, the rotation of GFP may be disturbed by its association with the partially narrow structure of crowders. These results suggest that the ratio between relative rotational diffusion time and relative translational diffusion time can be useful to characterize the MMC environment or structure.

Some macromolecules, including PEG, show a structural phase shift that depends on their concentration^34^. They form a blob with a lower concentration than its overlap concentration c* and an entangled network structure with higher concentration. Pol-FCS was performed on PEG6,000 to distinguish the phase shift, as shown in Fig. 4a. The overlap concentration of PEG6,000 was estimated as c* = 79 mg/mL based on an earlier calculation^32^. The plot shows a smaller slope at a concentration range smaller than 74 mg/mL and a larger slope in the higher range. In the lower concentration range, GFP could freely rotate among PEG6,000 blobs when compared with the high concentration range (see Fig. 4b). The relatively larger slope in the high concentration range likely indicates that the rotation of GFP was disturbed by the entangled network-like structure of PEG6,000 (see Fig. 4c). Thus, it is possible for Pol-FCS to detect the phase shift in the macromolecule solution at the overlap concentration. Supplementary Fig. 2 shows the relationship between the relative translational or rotational diffusion and the PEG6,000 concentration. It is difficult to determine the overlap concentration from only the translational diffusion measurement. However, the measured rotational diffusion plateaued around the overlap concentration. This might indicate an intermediate state between the two phases of PEG6,000. Similarly, the ratio between the relative rotational and translational diffusion time (see Supplementary Fig. S2c) plateaued in concentration range more than 50 mg/mL indicating that the rotational and rotational diffusion of GFP in the entangled network-like structure of PEG6,000 including the intermediate state were likely disturbed same degree.

Furthermore, the possibility of analyzing the MMC condition in living cells and its application was demonstrated (Fig. 5). The standard deviations of the results were larger than the measurement of solutions. This is likely caused by the high heterogeneity of environment within single cell as reported by Dross et al.^35^ and individuality of cells. Pol-FCS measurements successfully revealed the difference of nano-environment of each cell lines for GFP-sized molecules. The results also showed that the difference in the nano-environment of cytosol disappeared with nocodazole treatment. This likely indicates that the difference in the microtubule network of each cell line is one reason for the difference in the nano-environment, and the difference was cancelled by depolymerization of microtubules due to nocodazole treatment. This was surprising because the network of microtubules is not too fine to disturb GFP diffusion on the geometrical scale of the confocal region. The difference among each cell line before nocodazole treatment might be due to an indirect effect due to relatively large proteins, organelles, and other structures whose diffusion is restricted by the microtubule network. It should be noted that the nocodazole treatment could be an artefact because such the drug treatment generally stresses cells in addition to microtubule disassembly.

Next, we compared the differences in the nano-environment in different cell cycles. The results are shown in Fig. 6. The relative translational diffusion time was 3–4, and the relative rotational diffusion time was 2–2.5. This is similar to the results obtained from a 150–200 mg/mL ficoll solution. This likely indicates that the ficoll solution is a good MMC solution that mimics the rotational and translational diffusion in cells for GFP. In addition, the translational diffusion of GFP in the cytosol and nucleus were changed by cell synchronization. On the other hand, the rotational diffusion was not changed. This likely indicated that there were more crowded in both the cytosol and the nucleus in S-phase, however, the environment within the radius of gyration of GFP was not changed. It is possible that the affinity and shape of proteins in cells is controlled by the MMC condition change because of the cell cycle. Further experiments on several different cell cycles are needed to discuss the cell cycle dependency on MMC conditions. However, Pol-FCS measurement for every cell cycle is relatively difficult because the GFP concentration in the M phase is too high for Pol-FCS measurement. To achieve Pol-FCS measurement for every cell cycle, an improved method for GFP expression or other fluorescent probes is necessary.

Thus, the ratio between the relative rotational and translational diffusion times is very useful for characterizing the nano-environment, including MMC, macromolecular gels, and live cells. Especially in highly heterogeneous samples, Pol-FCS measurement is one of the best tools because the translational diffusion and rotational diffusion of probe molecules can be simultaneously evaluated. Also, the measurement can be performed separately in different cell compartments because the measurement region is much smaller than cells because of confocal detection.

However, the effect of molecular diffusion from the surrounding environment would not be identical for all molecules. To adequately understand the nano-environment in living cells, further Pol-FCS measurement using multiple size probes is necessary because the nano-environment effect would depend on the size of the probe molecules, as discussed in an earlier study^4^. In the future, a more detailed analysis of the MMC environment will be available if appropriate probes with different sizes are found.

Furthermore, Pol-FCS may be useful for evaluating other nano-environments such as liquid–liquid phase separation^36^ of proteins in living cells because proteins are highly condensed in environments such as the MMC environment.

## Materials and methods

### Polarization-dependent FCS

In this study, a Pol-FCS system modified from our existing system^21^ was used. Supplementary Fig. 1 shows a schematic diagram of the newly constructed Pol-FCS system. An excitation laser with a wavelength of 488 nm (LP488-SF20, Thorlabs) was collimated, polarized by a polarizer (PL), and reflected by a dichroic mirror (DM) (Di02-R488-25 × 36, Semrock, USA). The laser was focused into a fluorescent solution sample using an objective lens (OL) (Uplan Apo 60× 1.20W, Olympus, Japan).

Fluorescence emitted only from the sample passed through the DM and an emission filter (BLP01-488R-25, Semrock, USA), and was split into two directions by a polarization beam splitter. Fluorescence with parallel polarization to excitation was reflected by the PBS, and fluorescence with cross-polarization passed through the PBS. Each polarized fluorescence was split in two directions by beam splitters (BS_1_ and BS_2_). Finally, the fluorescence was detected by four avalanche photodiodes (D_1_–D_4_) through multimode optical fibers MM_1_–MM_4_. The aperture of each core of the multimode optical fibers played a role of pinhole. The core diameter of the multimode optical fibers was 50 µm. This was corresponding to 1.68 Airy unit in our system. The positions of MM_1_–MM_4_ were adjusted to detect maximum fluorescence intensity with the sample of 100 nM rhodamine 6G solution in advance of experiment. In this study, the outputs from D_1_ and D_2_ were connected to a hardware correlator (Flex02-01D, Correlator.com, USA), and a cross-correlation function (CCF) was obtained. This measurement corresponds to the X-XX condition of Pol-FCS in an earlier study^21^. The samples were putted on the sample stage of a fluorescence microscope (Axiovert 100 TV, Zeiss, Germany) to determine the measurement position precisely.

In the case of highly concentrated MMC solutions, the measurement volume of Pol-FCS can be deformed because of refractive index mismatch. Such a deformation of the measurement volume could affect the translational diffusion time. To avoid such artifacts, Pol-FCS measurements were performed near the coverslip of glass bottom dishes or 8-well chambers. The measurement point was approximately 5 μm from the surface of the coverslip. The effect of refractive index mismatch on FCS measurement is shown in Supplementary Fig. 3. The black broken line show reference line obtained from relative viscosity of sucrose solution at 30 °C, which was calculated by a reported value^37^.

All the measurements in this work were performed at room temperature maintained at 24–26 °C. It should be noted that the experiments at the temperature could be a cause of stress for cells.

### Pol-FCS measurement

The samples were placed on a glass bottom dish (D11140H, MATSUNAMI GLASS, Japan) or on the chambers of an 8-well chamber slide (Nunc Lab-Tek Chamber Slide System, Thermo Fisher Scientific, USA) for the measurement. Using Eq. (3) and the reported diffusion coefficient of GFP (87 µm^2^/s), the lateral radius of the focal region was obtained as 0.25 μm. The excitation laser power was 0.7–11.0 μW (0.4–5.6 kW/cm^2^) at the focal plane of the OL. The 30-s measurement was repeated 10 times for MMC solutions. As a result, one averaged CCF was obtained from each 10 times measurement for following fitting analysis. The 5-s measurement was repeated 20 times for GFP-expressing cells. One averaged CCF was obtained from each 20 times measurement for following fitting analysis. The cells were prepared at the same day for the same cell line in more than two dishes.

The obtained CCF was analyzed by the nonlinear least squares method without weighting using the following model equations for Pol-FCS^21,27^:

8 $$\mathrm{G}\left(\uptau \right)={G}_{D}\left(\tau \right) \cdot {G}_{R}\left(\tau \right)\cdot {G}_{T}\left(\tau \right)$$

9 $${G}_{D}\left(\tau \right)=\frac{1}{N}{\left(1+\frac{\tau }{{\tau }_{\mathrm{trans}}}\right)}^{-1}{\left(1+\frac{\tau }{{{s}^{2}\cdot \tau }_{\mathrm{trans}}}\right)}^{-\frac{1}{2}}$$

10 $${G}_{R}\left(\tau \right)=1+{F}_{\mathrm{rot}}\mathrm{exp}\left(-\frac{\tau }{{\tau }_{\mathrm{rot}}}\right)$$

11 $${G}_{T}\left(\tau \right)=1+\frac{{F}_{\mathrm{T}}}{1-{F}_{\mathrm{T}}}\mathrm{exp}\left(-\frac{\tau }{{\tau }_{\mathrm{T}}}\right)$$

where *G*_D_, *G*_R_, and *G*_T_ are the components of translational diffusion, rotational diffusion, and relaxation of photodynamical process, respectively. *τ*_trans_ and *τ*_rot_ are the relaxation times for translational diffusion and rotational diffusion, also called the translational diffusion time and the rotational diffusion time, respectively. Longer *τ*_trans_ or *τ*_rot_ imply slower translational or rotational diffusion. *F*_rot_ is a fraction of the rotational diffusion component. The structure parameter *s*, which is the ratio of the lateral radius to the axial radius of the measurement volume (focal volume), is fixed at 5.0 in the nonlinear curve fitting analysis of this study. *τ*_T_ and *F*_T_ are the relaxation time of photodynamical process and the fraction of that component, respectively. The relaxation time of photodynamical process of GFP was measured in PBS solution as τ_T_ = 1.9 μs, and the value was fixed in another experiment to avoid misfitting the component fitted as a rotational diffusion component. The component of the photodynamical process might be the component due to the transition to the triplet state, the transition to a non-fluorescent state induced by conformational changes, and isomerization. According to a detailed report about the photodynamic properties of GFP by Widengren et al., the measured *τ*_T_ corresponded well to the component due to the transition to a non-fluorescent state^27^. The fitted curve was well fitted without any other photodynamical process, as shown in Fig. 1a. Also, fractions of the components due to the possible photodynamical process of GFP were constant for our excitation laser power range (0.4–5.6 kW/cm^2^)^27^.

### Macromolecular gels

*N*-(Carboxymethyl)-*N*,*N*-dimethyl-2-(methacryloyloxy) ethanaminium inner salt (CDME) was provided by Osaka Organic Chemical Industry (Japan). *N*,*N'*-Methylenebis(acrylamide) (MBAA) and 2-oxoglutaric acid were purchased from Wako (Japan).

Poly-*N*-(carboxymethyl)-*N*,*N*-dimethyl-2-(methacryloyloxy) ethanaminium inner salt (pCDME) gels^28,38^ were synthesized through radical polymerization. Aqueous solutions of 3.0 M of CDME as monomer, 3, 9, 18, or 30 mM of MBAA as crosslinker, and 3 mM of 2-oxoglutaric acid as initiator were prepared. Each solution was poured into a mold made of two glass plates and a silicone spacer (1 mm-thick), and was irradiated with UV light (365 nm) for 8 h in an argon glove box. The resulting pCDME hydrogels were washed with large volume of Milli-Q water four times for 36 h, and then soaked in PBS for 1 day.

Mesh sizes, ξ, of the pCDME gels were roughly estimated as: ξ ≈ ν^−1/3^ ≈ (*E*/3*k*_B_*T*)^−1/3^, where ν, *E*, *k*_B_, and *T* are the number of network strands per unit volume, Young’s modulus of the gel, Boltzmann constant, and absolute temperature, respectively^28^. The Young’s moduli, *E*, of the swollen pCDME gels synthesized from the feed crosslinker (MBAA) concentrations of 3, 9, 18, or 30 mM were characterized from tensile test^39^ as *E* = 10.8(± 0.7), 34.9(± 3.0), 74.6(± 4.4), and 162.0(± 11.6) kPa (*n* = 3–4), respectively; hence the mesh sizes were estimated as ξ = 10.5, 7.1, 5.5, and 4.2 nm, respectively.

The gels were soaked in 100 nM GFP solution in PBS for more than 3 days. They were cut to an appropriate size and placed on a glass bottom dish, and the Pol-FCS measurements of the GFP were performed inside them.

### MMC solutions

Glycerol (Wako, Japan), sucrose (Nacalai Tesque, Japan), ficoll (Nacalai Tesque, Japan), polyethylene glycol of 200 (PEG200) and 6,000 (PEG6,000) in molecular weight (Wako, Japan), and BSA (Sigma-Aldrich, USA) were used as purchased without purification. PBS solutions of each crowder with arbitrary concentrations were prepared as MMC solutions. GFP in PBS was added to the MMC solutions (the final concentration of GFP was 100 nM).

### Cell preparation for nocodazole treatment

HeLa, HEK293, N2a, and COS-7 cells were cultured in Dulbecco’s modified Eagle’s medium (DMEM) (Sigma-Aldrich, USA) at 37 °C with 5% CO_2_.

1.0 μg of plasmid pmGFP was transfected into cells (5 × 10^5^ cells) using Lipofectamine 2000 (Life Technologies, USA).

After incubation for 24 h, the medium was replaced with fresh medium, and nocodazole was added up to a final concentration of 25 µM. After incubation in a fridge for 30 min at 5 °C, the medium was replaced with Opti-MEM I (Thermo Fisher Scientific, USA), and nocodazole was added up to a final concentration of 25 µM.

### Cell preparation for cell synchronization

HeLa cells were cultured in Dulbecco’s modified Eagle’s medium (DMEM) (Sigma-Aldrich, USA) at 37 ℃ with 5% CO_2_.

1.0 μg of plasmid pmGFP and 0.9 μg of plasmid pCAGGS were transfected into HeLa cells (5 × 10^5^ cells) using Lipofectamine 2000 (Life Technologies, USA).

After incubation for 24 h, cell synchronization using the double thymidine block method was applied to arrest cells in the early S phase. Thymidine (Sigma-Aldrich, USA) was added to the medium upto a final concentration of 2 mM and incubated for 14 h at 37 °C with 5% CO_2_. The cells were washed twice with 2 mL of PBS. Then, 2 mL of medium was added and incubated for 9 h at 37 °C with 5% CO_2_. Following incubation, thymidine was added to the medium upto a final concentration of 2 mM and incubated for 14 h at 37 °C with 5% CO_2_. Finally, the medium was replaced with 2 mL of Opti-MEM I (Thermo Fisher Scientific, USA) with thymidine (final concentration of 2 mM).

## Supplementary Information

Supplementary Information.

## Data Availability

All data generated or analyzed during this study are included in this published article (and its Supplementary Information files).
