# Supplementary material for: Quantitative evaluation of macromolecular crowding environment based on translational and rotational diffusion using polarization dependent fluorescence correlation spectroscopy
Source: Sci Rep. 2021 May 19;11:10594. doi: 10.1038/s41598-021-89987-7 (PMC8134472; doi:10.1038/s41598-021-89987-7)
Supplement: Supplementary file 1 — Supplementary Information. [file 41598_2021_89987_MOESM1_ESM.pdf]

Quantitative evaluation of macromolecular crowding environment based on translational and rotational diffusion using polarization dependent fluorescence correlation spectroscopy

Johtaro Yamamoto[1, *], Akito Matsui[2], Fusako Gan[2], Makoto Oura[2], Riku Ando[3], Takahiro Matsuda[4], Jian Ping Gong[4,5,6], and Masataka Kinjo[2]

[1] Bioimaging Research Group, Health and Medical Research Institute, National Institute of Advanced Industrial Science and Technology (AIST), Tsukuba, 305-8566, Japan

[2] Laboratory of Molecular Cell Dynamics, Faculty of Advanced Life Science, Hokkaido University, Sapporo, 001-0021, Japan

[3] Graduate School of Life Science, Hokkaido University, Sapporo, 001-0021, Japan

[4] Laboratory of Soft & Wet Matter, Faculty of Advanced Life Science, Hokkaido University, Sapporo, 001-0021, Japan

[5] Institute for Chemical Reaction Design and Discovery (WPI-ICReDD), Hokkaido University, Sapporo 001-0021 Japan

[6] Global Institution for Collaborative Research and Education (GI-CoRE), Hokkaido University, Sapporo 001-0021 Japan

[*] yamamoto-jtr@aist.go.jp

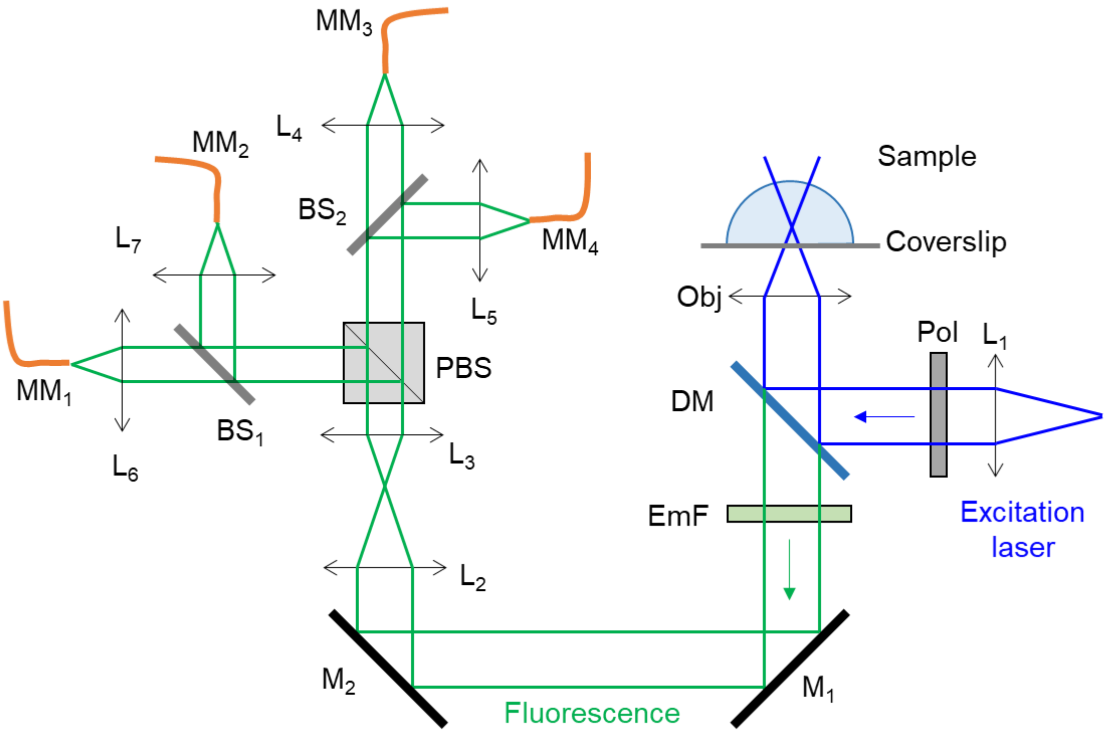

Supplementary Figure S1. Schematic diagram of experimental setup of Pol-FCS. L: Lens, M: Mirror, Pol: Polarizer, DM: Dichroic mirror, Obj: Objective lens, EmF: Emission filter, PBS: Polarizing beam splitter, BS: Pellicle beam splitter, MM: Multimode optical fiber, D: Avalanche photo diode.

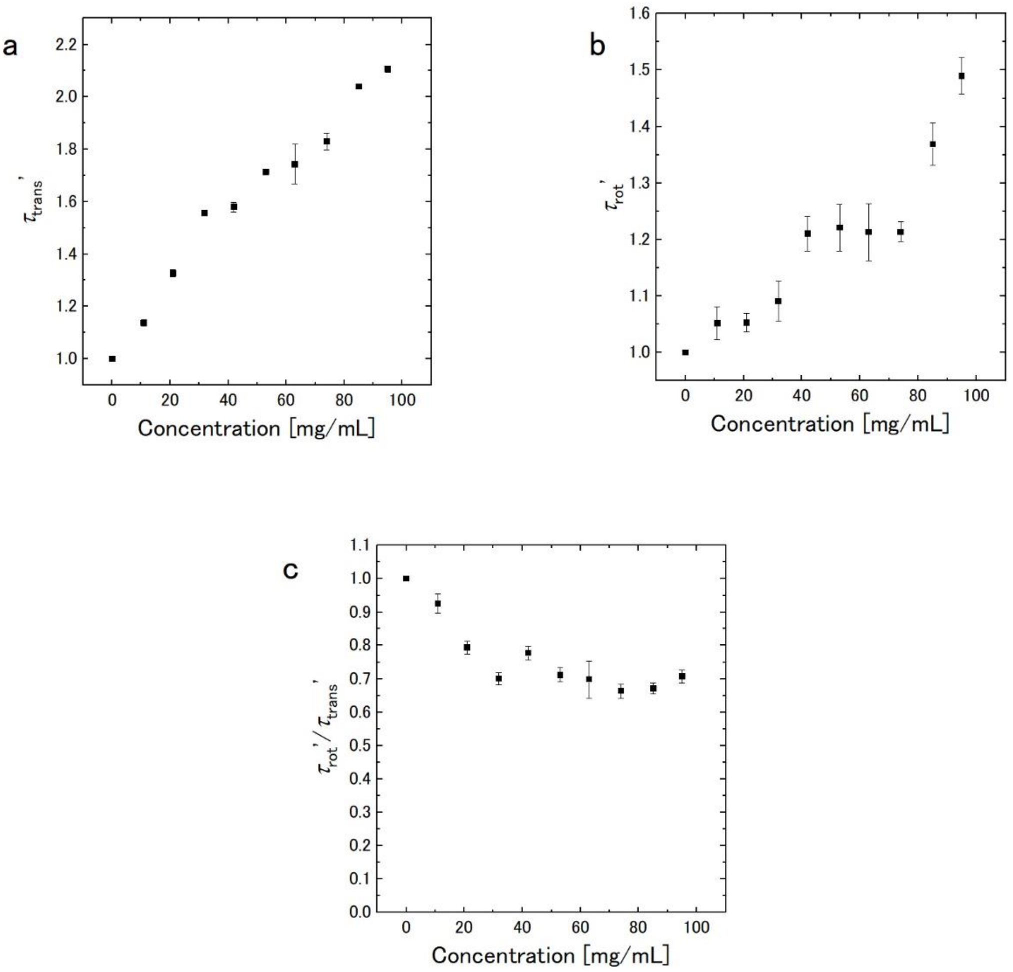

Supplementary Figure S2. Sucrose concentration dependency on the relative translational/rotational diffusion time of GFP in PBS solution. (a) Relationship between sucrose concentration and the relative translation diffusion time. (b) Relationship between sucrose concentration and the relative rotational diffusion time. (c) Relationship between sucrose concentration and the ratio of the relative rotational/translational diffusion time. The error bar shows the standard deviation (n = 3).

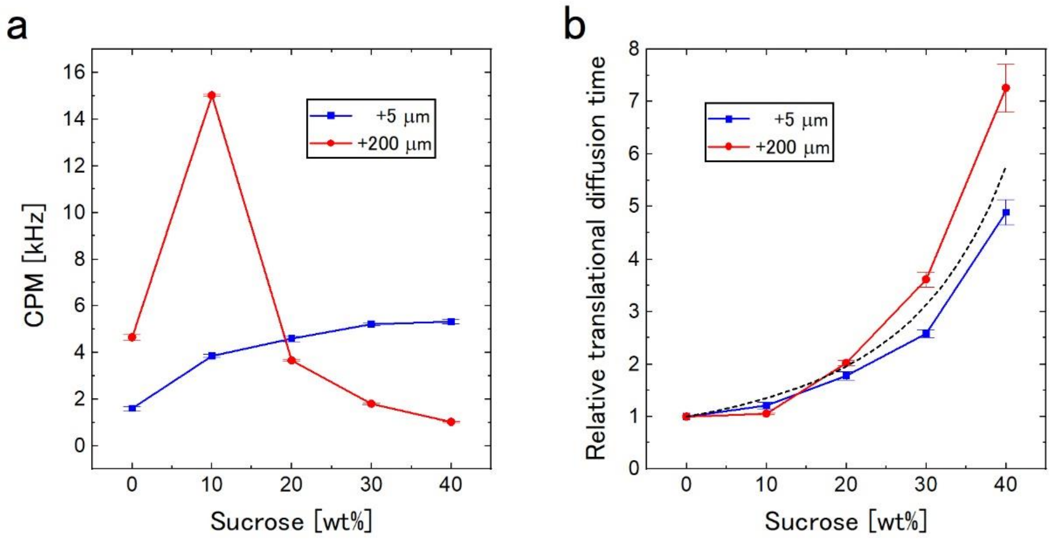

Supplementary Figure S3. Comparison of FCS measurement on GFP in sucrose solution at the different measurement positions. The sucrose was dissolved in PBS. The experiment was performed using LSM510 with ConfoCor3 system (Carl Zeiss, Germany). (a) Counts per molecule (CPM), which is the brightness of a single GFP measured by FCS. At +200 μm distance from the coverslip, the effect of the aberration due to refractive index mismatch was more extensive than that at +5 μm. This indicated the deformation of the confocal volume was more suppressed near the coverslip than the position far from the cover slip. If the aberration was perfectly corrected by a correction ring of the objective lens, the CPM should take maximum value at 0wt% sucrose. However, the CPM took the highest value at minimum of correction ring in 0wt% sucrose solution. This imperfect correction of the aberration by correction ring may cause the decrease of CPM and elongation of diffusion time, however, that is not a problem for FCS analysis if CPM is not too low. (b) Relative translational diffusion time normalized by the diffusion time in the 0wt% sucrose solution (PBS). The black broken line show reference line obtained from relative viscosity of sucrose solution at 30°C, which was calculated by a reported value [37]. The difference between the reference line and the result of +5 μm was smaller than it of +200 μm. The duration for a single measurement was 10 s × 10 loops. The measurements were repeated 5 times on the same sample (n = 5). The excitation laser power was 3.6 μW. The error bars show the standard deviation.

Supplementary Table S1. Relative translational/rotational diffusion time in cytosol of different cell lines without nocodazole treatment. Values are average ± standard deviation.

	HeLa (*n* = 19)	HEK293 (*n* = 15)	N2a (*n* = 20)	COS-7 (*n* = 14)
τ'_{trans}	3.49 ± 1.00	2.00 ± 0.49	2.85 ± 0.74	2.31 ± 0.27
τ'_{rot}	2.03 ± 0.34	2.38 ± 0.51	2.48 ± 0.66	2.04 ± 0.26

Supplementary Table S2. Relative translational/rotational diffusion time in cytosol of different cell lines with nocodazole treatment. Values are average ± standard deviation.

	HeLa (*n* = 15)	HEK293 (*n* = 17)	N2a (*n* = 15)	COS-7 (*n* = 17)
τ'_{trans}	2.71 ± 0.37	2.39 ± 0.42	2.80 ± 0.74	2.01 ± 0.47
τ'_{rot}	2.26 ± 0.35	1.95 ± 0.41	2.08 ± 0.49	2.06 ± 0.23

Supplementary Table S3. *p*-values of Welch's *t*-test on the relative translational diffusion time of GFP in cytosol without nocodazole treatment between each cell lines. * and ** indicate $p<0.05$ and $p<0.01$, respectively.

	HeLa	HEK293	N2a	COS-7
HeLa		4.80×10^{-6} **	0.029 *	7.25×10^{-5} **
HEK293	4.80×10^{-6} **		2.88×10^{-4} **	0.046 *
N2a	0.029 *	2.88×10^{-4} **		0.006 **
COS-7	7.25×10^{-5} **	0.046 *	0.006 **	

Supplementary Table S4. *p*-values of Welch's *t*-test on the relative rotational diffusion time of GFP in cytosol without nocodazole treatment between each cell lines. * and ** indicate $p<0.05$ and $p< 0.01$, respectively. n.s. indicate there was no significant difference.

	HeLa	HEK293	N2a	COS-7
HeLa		0.030 *	0.012 *	0.930 n.s.
HEK293	0.030 *		0.627 n.s.	0.030 *
N2a	0.012 *	0.627 n.s.		0.012 *
COS-7	0.930 n.s.	0.030 *	0.012 *	

Supplementary Table S5. *p*-values of Welch's *t*-test on the relative translational diffusion time of GFP in cytosol with nocodazole treatment between each cell lines. * and ** indicate $p<0.05$ and $p<0.01$, respectively. n.s. indicate there was no significant difference.

	HeLa	HEK293	N2a	COS-7
HeLa		0.028<br>*	0.687<br>n.s.	0.184<br>n.s.
HEK293	0.028<br>*		0.072<br>n.s.	0.463<br>n.s.
N2a	0.687<br>n.s.	0.072<br>n.s.		0.154<br>n.s.
COS-7	0.184<br>n.s.	0.463<br>n.s.	0.154<br>n.s.	

Supplementary Table S6. *p*-values of Welch's *t*-test on the relative rotational diffusion time of GFP in cytosol with nocodazole treatment between each cell lines. * and ** indicate $p<0.05$ and $p<0.01$, respectively. n.s. indicate there was no significant difference.

	HeLa	HEK293	N2a	COS-7
HeLa		0.027<br>*	0.247<br>n.s.	0.076<br>n.s.
HEK293	0.027<br>*		0.435<br>n.s.	0.315<br>n.s.
N2a	0.247<br>n.s.	0.435<br>n.s.		0.933<br>n.s.
COS-7	0.076<br>n.s.	0.315<br>n.s.	0.933<br>n.s.	

Supplementary Table S7. *p*-values of Welch's *t*-test on the change of relative translational and rotational diffusion time of GFP by nocodazole treatment. * and ** indicate $p<0.05$ and $p<0.01$, respectively. n.s. indicate there was no significant difference.

	HeLa	HEK293	N2a	COS-7
τ'_{trans}	0.005<br>**	0.023<br>*	0.854<br>n.s.	0.558<br>n.s.
τ'_{rot}	0.064<br>n.s.	0.013<br>*	0.045<br>*	0.775<br>n.s.

Supplementary Table S8. Relative translational diffusion time in HeLa cells. * and ** indicate $p<0.05$ and $p<0.01$, respectively. n.s. indicate there was no significant difference.

Cell synchronization	Cytosol (Cyt.)	Nucleus (Nuc.)	*p* (Cyt. vs Nuc.)
No ($n = 22$)	3.00 ± 0.70	3.01 ± 0.56	0.959<br>n.s.
S-phase ($n = 20$)	3.74 ± 0.50	3.87 ± 0.65	0.483<br>n.s.
p (No vs S-phase)	3.10×10^{-4}<br>**	5.06×10^{-5}<br>**	

Supplementary Table S9. Relative rotational diffusion time in HeLa cells. * and ** indicate $p<0.05$ and $p<0.01$, respectively. n.s. indicate there was no significant difference.

Cell synchronization	Cytosol (Cyt.)	Nucleus (Nuc.)	*p* (Cyt. vs Nuc.)
No ($n = 22$)	2.17 ± 0.54	2.16 ± 0.55	0.952<br>n.s.
S-phase ($n = 20$)	2.48 ± 0.59	2.37 ± 0.73	0.603<br>n.s.
p (No vs S-phase)	0.085<br>n.s.	0.303<br>n.s.	
